# Supplementary figures and images for: Inactivation of EMILIN-1 by Proteolysis and Secretion in Small Extracellular Vesicles Favors Melanoma Progression and Metastasis
Source: Int J Mol Sci. 2021 Jul 9;22(14):7406. doi: 10.3390/ijms22147406 (PMC8303474; doi:10.3390/ijms22147406)

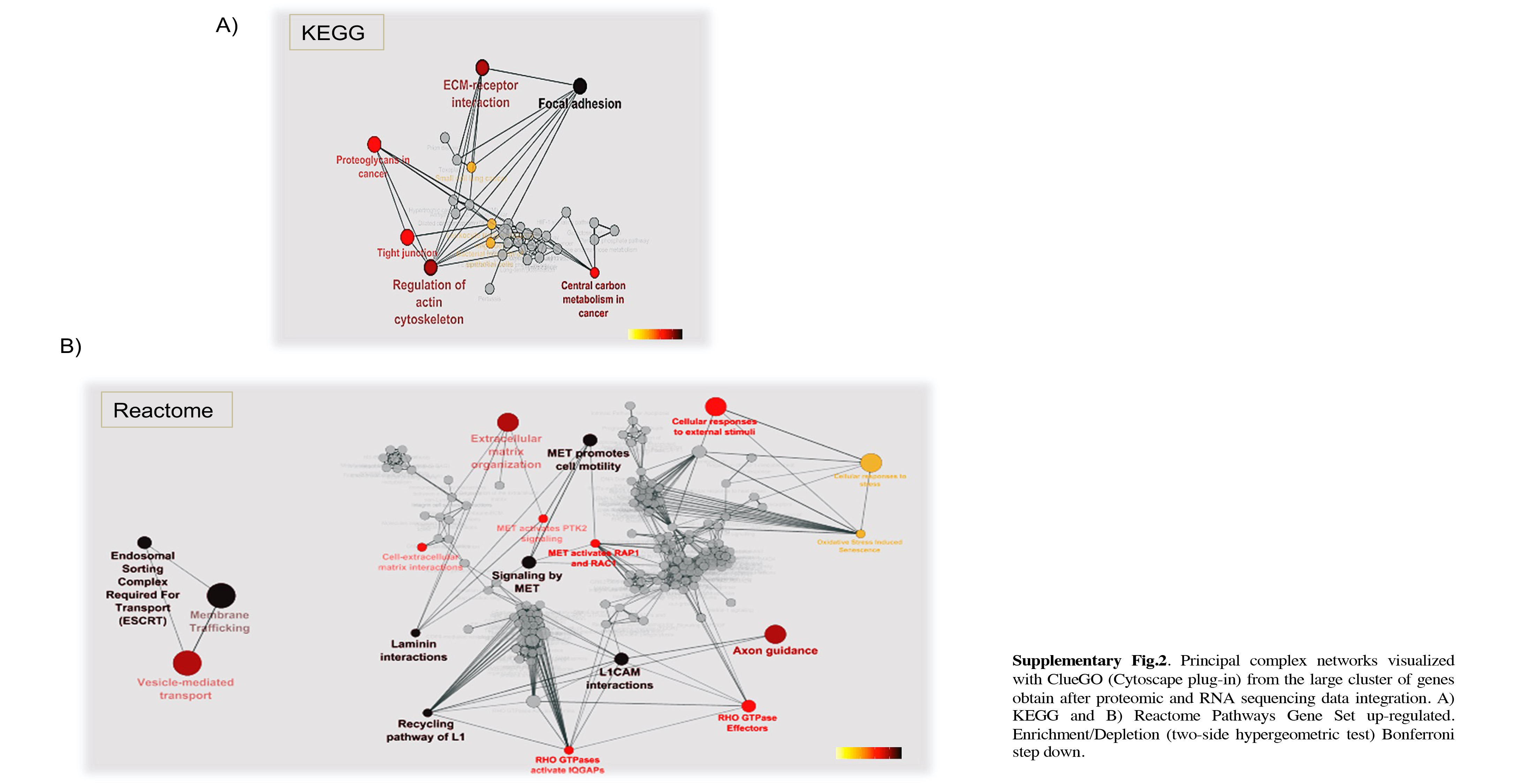

Supplement: Supplementary file 1 [file ijms-22-07406-s001.zip › Supl. Fig 2.tif]

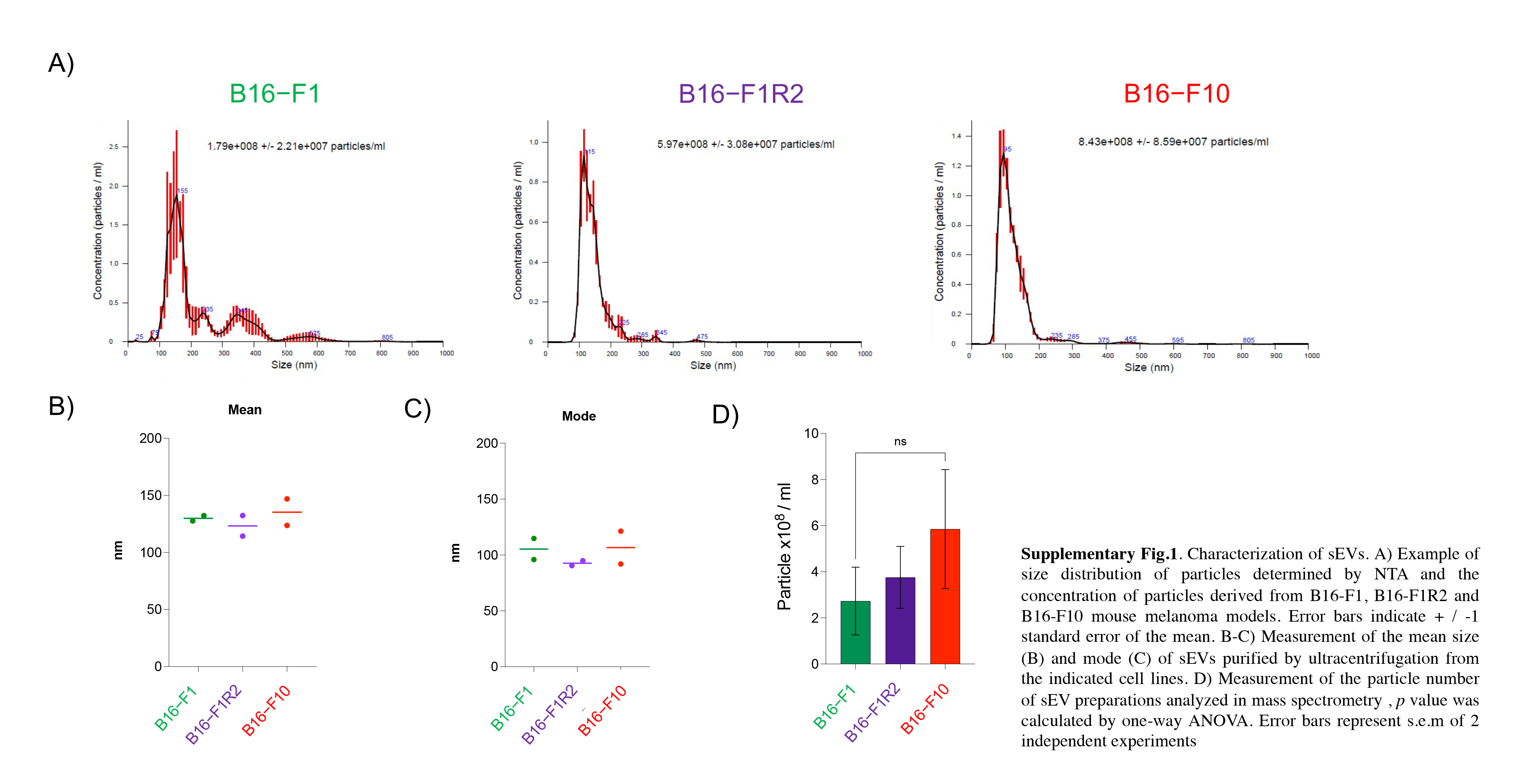

Supplement: Supplementary file 1 [file ijms-22-07406-s001.zip › Suppl Fig 1.tif]

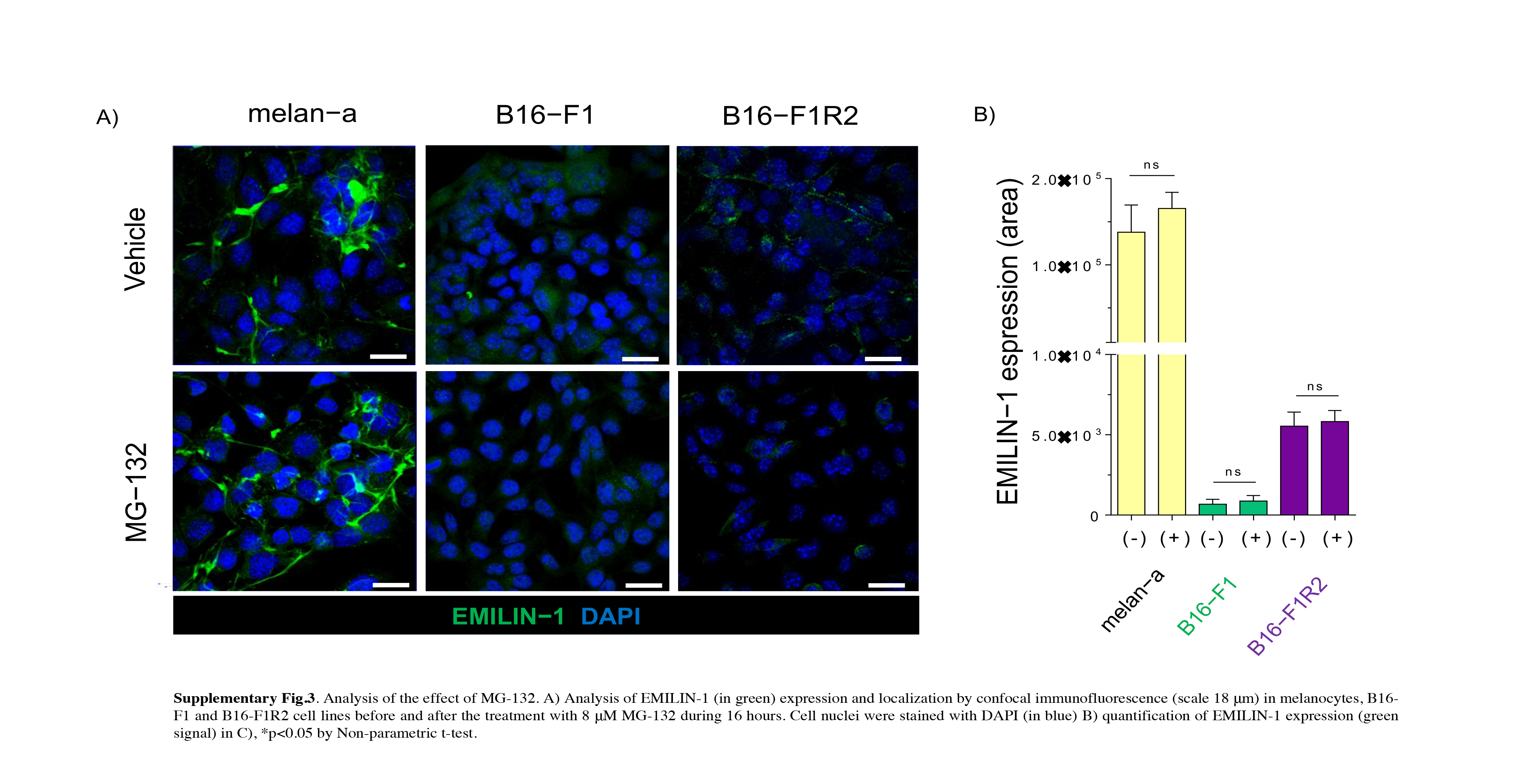

Supplement: Supplementary file 1 [file ijms-22-07406-s001.zip › Suppl. Fig 3.tif]
